# Identifying climatic drivers of respiratory syncytial virus (RSV) seasonality in Antananarivo, Madagascar, 2011–2021: a sentinel surveillance study

**DOI:** 10.1136/bmjph-2024-001093

**Published:** 2024-09-23

**Authors:** Tsiry Hasina Randriambolamanantsoa, Norosoa Harline Razanajatovo, Hafaliana Christian Ranaivoson, Laurence Randrianasolo, Hasina Joelinotahiana Rabarison, Helisoa Razafimanjato, Arvé Ratsimbazafy, Danielle Aurore Doll Rakoto, Jean-Michel Heraud, Vincent Lacoste, Cara E Brook

**Affiliations:** 1 National Influenza Center, Virology Unit, Institut Pasteur de Madagascar, Antananarivo, Madagascar; 2 Department of Ecology and Evolution, University of Chicago, Chicago, Illinois, USA; 3 Department of Zoology and Animal Biodiversity, University of Antananarivo, Antananarivo, Madagascar; 4 Doctoral School of Life and Environment Sciences, Faculty of Sciences, University of Antananarivo, Antananarivo, Madagascar

**Keywords:** Epidemiology, Public Health, Sentinel Surveillance

## Abstract

**Introduction:**

Respiratory syncytial virus (RSV) is a primary source of acute lower respiratory tract infection, the leading cause of death in children under 5. Over 99% of RSV-attributed deaths occur in low-income countries, including Madagascar. RSV transmission is linked to climate, driving highly seasonal dynamics.

**Methods:**

We used generalised additive models (GAMs) to identify correlates of reported RSV infections in Antananarivo, Madagascar, from January 2011 to December 2021, then fit catalytic models to cumulative age-structured incidence to estimate age-specific force of infection (FOI). We fit a time-series susceptible-infected-recovered (TSIR) model to the dataset to estimate weekly RSV transmission, then evaluated associations with precipitation, humidity and temperature using generalised linear models. We used GAMs to quantify interannual trends in climate and assess whether significant deviations in RSV burden occurred in years representing climatic anomalies.

**Results:**

Reported RSV infections in Antananarivo were significantly associated with patients aged ≤2 years. Highest FOI was estimated in patients aged ≤1 year, with transmission declining to near-zero by age 5 before rising in older (60+) cohorts. TSIR models estimated a January to February peak in RSV transmission, which was strongly positively associated with precipitation and more weakly with temperature but negatively related to relative humidity. Precipitation, humidity and temperature all increased across the study period in Antananarivo, while reported RSV infections remained stable. Significant deviations in RSV burden were not associated with clear climate anomalies.

**Conclusions:**

Stable rates of reported RSV infections in Antananarivo across the past decade may reflect contrasting impacts of elevated precipitation and increased humidity on transmission. If future climate changes yield more rapidly accelerating precipitation than humidity, this could accelerate RSV burden. Introduction of recently developed public health interventions to combat RSV in low-income settings like Madagascar is essential to mitigating disease burden, in particular to combat any future climate-driven increases in transmission or severity.

**WHAT IS ALREADY KNOWN ON THIS TOPIC**

- Respiratory syncytial virus (RSV) is an important driver of acute lower respiratory tract infections, which represent the leading cause of mortality in children under 5 across the globe. RSV demonstrates highly seasonal dynamics, as its transmission is linked to climate.

**WHAT THIS STUDY ADDS**

- We quantified correlates of RSV infection and estimated the seasonal transmission rate for RSV from reported patient data in Antananarivo, Madagascar. We found that RSV transmission is primarily concentrated in very young children (≤1 year) in Antananarivo and positively associated with high precipitation and low humidity, which focus most transmission in Madagascar’s January to February rainy season.

**HOW THIS STUDY MIGHT AFFECT RESEARCH, PRACTICE OR POLICY**

- Our study suggests that RSV burden may intensify with future climate change, particularly higher rainfall. We emphasise the high public health importance of accelerating the introduction of recently developed monoclonal antibody and vaccination interventions to combat RSV in low-income settings like Madagascar.

## Introduction

 Respiratory syncytial virus (RSV) is a highly contagious virus that primarily infects infants and young children. Worldwide, most children contract RSV before reaching 2 years of age.^1^ The virus infects the upper and lower respiratory tracts causing infections that range from common cold to bronchiolitis and pneumonia. Globally, acute lower respiratory tract infections (ALRTIs) are the leading cause of death in children under 5 (CU5),^2^ and approximately one-fourth of ALRTIs are estimated to be attributable to RSV each year.^3 4^ Over 99% of CU5 fatalities from RSV-related ALRTIs occur in low-income countries.^3 4^ RSV can also affect older age groups and those immunocompromised or with comorbidities such as asthma and other lung diseases, diabetes and heart failure.^5 6^ In Madagascar, RSV remains the most common cause of ALRTI and a major cause of hospital admission in CU5.^7^ It is estimated that approximately 11 299 hospitalisations per year can be attributed to RSV in CU5 in Madagascar.^8^ Three years (2010–2013) of laboratory surveillance from Institut Pasteur de Madagascar (IPM) aimed at identifying the aetiology of severe acute respiratory infections (SARI) showed that nearly 37.7% of samples were positive for RSV, with the vast majority of patients (81%) representing CU5.^7^ A further 38.9% of SARI surveillance samples collected between 2018 and 2022 in Madagascar additionally tested positive for RSV, again with the majority concentrated in CU5.^9^

Given the globally significant impact of RSV on childhood morbidity and mortality, RSV has been the subject of numerous research studies across many countries.^3 10 11^ Particular areas of focus have included standard reporting of disease burden, clinical features and at-risk groups for severe infections—typically aimed at defining best practices for the monitoring of RSV infection in CU5 and avoiding the misuse of antibiotics.^11^,^14^ In addition, considerable work has been devoted to quantification of seasonal transmission dynamics for RSV—in order to better predict the timing and magnitude of severe epidemics.^15^,^17^

The seasonality of RSV circulation shows different patterns depending on geographical location, though most localities are characterised by a clear annual peak in transmission. In temperate countries, peak cases generally predominate during the colder months—from September to January in the Northern Hemisphere and from March to June in the Southern Hemisphere.^18^ In tropical and subtropical countries, the timing of the peak RSV season is more variable across locations, with some studies reporting highest RSV activity during peak rains^19^,^21^ and others reporting elevated case loads during warmest months of the year.^22^ Madagascar has been previously documented as an anomaly in the study of seasonal circulation for respiratory viruses, particularly influenza, for which transmission is highly irregular.^23^ RSV circulation, however, is well defined, with burden concentrated in the first half of each calendar year and the peak in cases between February and March.^7 9^

Climate is known to impact RSV circulation across diverse geographical localities^3 17 23^ but has thus far remained largely unexplored in Madagascar. Prior work demonstrates a specific role for both precipitation and humidity in driving RSV transmission across the globe—with stronger effects of humidity observed in temperate regions with very dry winters but a more pronounced impact of precipitation in tropical localities that are on average more humid throughout the year.^17^ Because of this, we anticipated a significant role for rainfall in driving RSV transmission in highly humid Madagascar. Quantitative understanding of the climatic factors that drive intra-annual seasonality in RSV transmission is essential to predicting how RSV dynamics will respond to future interannual climatic changes. Here, we aimed to (1) identify statistical correlates of RSV infection from reported cases of respiratory infection in Antananarivo, Madagascar, over the past decade, (2) estimate variation in the age-structured force of infection (FOI) for RSV cases in Antananarivo, (3) quantify the seasonality of RSV transmission across the study period and (4) identify climatic variables associated with peak RSV transmission.

## Methods

### Study location and setting

The influenza sentinel surveillance network (ISSN) in Madagascar has been operational since 1978.^24 25^ The aim is to monitor the circulation of influenza viruses as well as other respiratory viruses of public health concern, such as RSV and, more recently, SARS-CoV-2. The ISSN comprised an influenza-like illness (ILI) surveillance programme that now involves 21 referral primary healthcare centres (Centre de Surveillance Biologique de Référence (CSB-R)) and a severe acute respiratory infection (SARI) surveillance programme in five selected hospitals. The ISSN is complemented via the monitoring of death certificate and mortality data collected from six districts in Antananarivo through the Bureau Municipal d’Hygiène recently renamed Direction de l’Eau, l’Assainissement et l’Hygiène.^26^

For the present study, we used data collected from sentinel sites in Antananarivo, the capital city of Madagascar, over 11 years (from January 2011 to December 2021). Antananarivo is home to around 3 million inhabitants^27^ and hosts a tropical climate profile that is hot and rainy in summer (November to April) and cold and dry in winter (May to October). Sites used in our analysis included three ILI sites: Ostie Behoririka (BHK, −18.90, 47.53), Centre de Santé Maternelle et Infantile de Tsaralalana (CSMI-TSL, −18.91, 47.53), Dispensaire Manjakaray (MJR, −18.89, 47.53); and two SARI sites: Centre Hospitalier de Soavinandriana (CENHOSOA, −18.90, 47.545) and Centre Hospitalier Universitaire Mère-Enfant Tsaralalàna (CHUMET, −18.91, 47.52). Respectively, each site serves a catchment population of 28 574 (BHK), 43 222 (CSMI-TSL), 8000 (MJR), 89 000 (CENHOSOA) and 43 222 people (CHUMET).^28 29^

### Study subjects

All patients presenting to focal sites with ILI or SARI symptoms were enrolled. Details on case definitions and enrolment procedures have been previously published. Briefly, ILI classification criteria refer to patients of any age reporting with (a) a recorded temperature ≥38°C or a history of fever and (b) a cough of ≤10 days duration, who (c) do not require hospitalisation. Patients with SARI report with these same symptoms but additionally require hospitalisation.^30^ For each enrolled patient, demographic, socioeconomic, clinical and epidemiological data were recorded in case report forms.

### Patient and public involvement

No patient and public involvement.

### Virus detection

Nasopharyngeal specimens were collected for each enrolled patient. After collection, samples were shipped daily to the Virology Unit at IPM where they were immediately processed or stored at 4°C until testing (a maximum of 2 days after collection, following WHO guidelines).^31 32^ All procedures for the processing of biological samples have been previously described.^33^ Briefly, nasopharyngeal swabs were screened for influenza A and B, RSV and, since March 2020, SARS-CoV-2, using discrete real-time reverse transcription-PCR assays (online supplemental text S1). Only the SARI sentinel site of CENHOSOA supplied samples consistently across the entire study period, while most sites supplied samples intermittently for shorter durations throughout the 2011–2021 time series (online supplemental figure S1).

### Climate data

Meteorological data reporting total precipitation (mm), mean relative humidity (%) and mean temperature (°C) at daily intervals in Antananarivo, Madagascar, across the study period were downloaded from the National Aeronautics and Space Administration database^34^ and summarised by week (summed for precipitation and averaged for humidity and temperature).

### Data analysis

#### RSV infection correlates from generalised additive models

We first aimed to identify correlates of RSV infection in patients reporting to study sites with ILI and SARI symptoms across the study period. To this end, we fit a series of generalised additive models (GAMs) in the binomial family, incorporating a response variable of RSV infection outcome (0/1, indicating negative or positive) for all patients with ILI and SARI tested in the dataset across all reporting sites.^35^ In the first GAM, we modelled all predictor variables as smoothing splines, with ‘day of year’ formatted as a cyclic cubic spline (to control for intra-annual seasonality in our dataset), ‘age’ formatted as a thin plate smoothing spline, and ‘sex’, ‘year’ and ‘reporting hospital’ formatted as factorial random effects (online supplemental table S1). We additionally fit a second GAM with ‘year’ formatted as a numerical thin plate smoothing spline to evaluate interannual patterns across the time series (online supplemental table S1). Finally, because age and sex data were only available for a subset (n=3242) of tested cases, we fit a third GAM in the binomial family to the full dataset of RSV test data (n=3432), incorporating only predictor variables of ‘day of year’ (as a cubic smoothing spline) and ‘year’ (as a random effect) (online supplemental table S1).

#### Estimation of age-structured FOI for reported RSV cases in Antananarivo

Building from age correlates of infection identified in GAMs, we next sought to quantify age-structured variation in the FOI for reported RSV cases across the study period. Methods for estimating FOI from age-stratified serological data for single-strain immunising pathogens are well established,^36^,^39^ and prior work has successfully adapted them for application to age-structured incidence data, in lieu of serology.^40^ Assuming individuals are born susceptible and immunity following infection is lifelong, the standard ‘catalytic’ model describes the cumulative probability p((a)) of encountering infection by age a as:

[1] $$P\left(a\right)=1-e^{-\int_{0}^{a}\lambda \left(a\right)da}$$

where $\lambda \left(a\right)$ is the age-specific FOI.

Following equation 1, we fit a series of catalytic models with variable number and duration of age classes to the cumulative incidence of reported cases of RSV by age in Antananarivo across the study period, then compared model fit to the data by Akaike information criterion (AIC) to evaluate the most appropriate number and distribution of age classes by which to segregate FOI (online supplemental table S2). We first evaluated a null model assuming a single constant FOI across all age classes, then tested increasingly complex models that specified disparate piecewise constant values for $\lambda \left(a\right)$ across age brackets (27 models tested in total; online supplemental table S2). We focused estimation of heterogeneity in $\lambda \left(a\right)$ on young and old patients presumed to be at heightened risk for RSV infection. Only models that achieved convergence in the fitting process were evaluated. All models were fit using a quasi-Newton (L-BFGS-B, limited memory Broyden–Fletcher–Goldfarb–Shanno algorithm) optimisation method in the ‘optim’ function of the base R (v 4.2.2) package ‘stats’; 95% CIs on the resulting age-specific FOI estimates were constructed from the Hessian matrix.

#### Seasonal transmission dynamics of RSV via time-series susceptible-infected-recovered modelling

We next sought to quantify the seasonality of RSV transmission across our study period using a time-series susceptible-infected-recovered (TSIR) modelling approach implemented in the R package, tsiR.^41^,^44^ One of the most widely used classes of the general SIR model, the TSIR model was originally developed to simplify the process of parameter estimation in fitting SIR models to time-series data for measles, a perfectly immunising, widespread childhood disease.^42^,^44^ More recently, TSIR has been adopted for application to other immunising childhood diseases, including varicella,^45^ rubella^46 47^ and RSV.^15 17^ TSIR depends on two critical assumptions, requiring (1) that over long time horizons, the sum of infected cases should be equivalent to the sum of births for highly infectious childhood diseases (for which all individuals are assumed to be eventually exposed), and (2) that the infectious period of the pathogen modelled is equal to the sampling interval for the time-series data. TSIR thus captures epidemic dynamics in a series of simple difference equations, whereby the susceptible population in a future timestep ($S_{t+1}$) can be modelled as:

[2] $$S_{t+1}={S_{t}+B}_{t}-I_{t}-\mu_{t}$$

where $B_{t}$ corresponds to birth inputs to the population in each timestep, $I_{t}$ indicates susceptibles lost to infection, $\mu_{t}$ is additive noise, and $S_{t}$ can be rewritten as $S_{t}={\overset{-}{S}+Z}_{t}$, where $\overset{-}{S}$ is the mean number of susceptibles in the population and $Z_{t}$ describes the (unknown) time-varying deviations around this mean. From this, the susceptible population can be reconstructed iteratively across a time series, after rewriting equation 2 in terms of $Z_{t}$ and the starting condition, $Z_{0}$:

[3] $$\sum_{k=0}^{t-1}B_{k}=-Z_{0}+\frac{\sum_{k=0}^{t-1}Ir_{k}}{\rho }+Z_{t}+\mu_{t}$$

Equation 3 assumes that ρ is the reporting rate of infection and $Ir_{k}$ is the reported incidence (in our case corresponding to reported cases of RSV in our study region). Because TSIR assumes no overlapping infection generations, the number of infections per timestep can then be expressed as the product of the susceptible and infected populations in the preceding generation, along with the time-varying transmission rate ($\beta_{t}$):

[4] $$I_{t+1}=\frac{\beta_{t}S_{t}I_{t}^{\alpha }}{N_{t}}$$

where the homogeneity parameter (α) corrects for epidemic saturation in the process of discretising a continuous epidemic. Equation 4 can then be easily log-linearised as:

[5] $$\ln I_{t+1}=\ln\beta_{t}+\ln\left(Z_{t}+\overset{-}{S}\right)+\mathrm{\alpha ln}I_{t}-lnN_{t}$$

allowing for estimation of $\beta_{t}$ via simple regression techniques.

From above, we first followed ref 17 to reconstruct the population susceptible to RSV in our catchment area in Antananarivo, Madagascar. To this end, we binned RSV positive cases by week across the time series for all reporting sites, and correspondingly summed the population catchments for each site over each week for which data were reported (eg, the population modelled was higher for timesteps reporting cases from a greater number of sites). To avoid errors in TSIR, we substituted a value of 1 for weeks reporting zero case. We estimated weekly total births for our study population across the time series by multiplying per capita publicly available birth rates for Madagascar reported by the World Bank^48^ against the summed population size of the catchment of all reporting study sites, divided evenly across weekly timesteps in a given year (per capita World Bank birth rates are reported as annual means only). Again, following ref 17, we reconstructed the susceptible population using a simple linear regression of cumulative cases against cumulative births (online supplemental table S3). We validated our susceptible reconstruction by comparing the coarse estimate of the basic reproduction number (R_0_) for RSV in Antananarivo that can be derived from the relationship $\overset{-}{S}=1/R_{0}$^41 46^ with that inferred from the mean birth rate and average age of infection across the time series, assuming $R_{0}=G/A$, where G is the inverse of the population birth rate and A is the average age of infection.^49^

Finally, following susceptible reconstruction, we implemented a log-link linear regression in the ‘Poisson’ family in the tsiR package to estimate the weekly RSV transmission rate (β). Though we modelled our data in 52 weekly timesteps per year, to avoid overfitting, we constrained our fitting to 26 distinct biweekly transmission rates across the 11-year dataset (eg, transmission was held constant between consecutive 2-week intervals; online supplemental table S3). Consistent with prior studies,^17 50^ we fixed the homogeneity parameter (α) at 0.97 in the fitting process; previous work has demonstrated that inference into seasonal transmission rates from TSIR is robust to the value of α.^51^

#### Climatic drivers of RSV transmission using generalised linear models

Following estimation of seasonal transmission rates for RSV in Antananarivo, we sought to quantify the impact of local climate variables on RSV transmission across the time series. Here, we used a generalised linear model (GLM) in the ‘gaussian family’, fit to the response variable of the natural log of the weekly RSV transmission rate, as estimated from TSIR, with fixed predictor variables of total weekly precipitation (mm), weekly mean relative humidity (%) and mean weekly temperature (°C) for Antananarivo across the study period. Our most comprehensive, global GLM thus took the following general form:

[6] $$\ln\left(Em{(\beta }_{t})\right)=b_{1}P_{t}+b_{2}\left(\frac{1}{H_{t}}\right)+b_{3}T_{t}$$

where $Em{(\beta }_{t})$ corresponds to the empirically estimated transmission rate from TSIR, and $P_{t}$, $H_{t}$ and $T_{t}$ correspond, respectively, to total weekly precipitation, mean weekly relative humidity and mean weekly temperature at time t. Using the ‘dredge’ function in the R package MuMIn,^52^ we then conducted model selection by comparing the relative corrected AIC (AICc) of all predictor variable combinations of the global model defined in equation 6 (online supplemental table S4). We used the fitted correlation coefficients of the top-performing model from this selection exercise to explore the predicted impact of climatic variation on RSV transmission. Initially, we also considered GLMs exploring lagged interactions between climate variables and transmission response; however, likely because RSV is a directly transmitted infection, these representations were not well supported by the data, and we eventually disregarded this approach.

#### Interannual trends in Antananarivo climate variables and RSV case counts from GAMs

Finally, we aimed to quantify interannual trends for all Antananarivo climate variables considered across the 2011–2021 time series, while controlling for intra-annual seasonal variation. To this end, we fit three separate climate GAMs in the ‘gaussian’ family to the response variables of, respectively, total weekly precipitation (mm), mean weekly relative humidity (%) and mean weekly temperature (°C) for Antananarivo. Each GAM incorporated a fixed, numerical effect of ‘year’ and cubic smoothing spline of ‘day of year’ (online supplemental table S5). We additionally fit a fourth GAM in the ‘Poisson’ family, which took the same form as the climate GAMs but included a response variable of total weekly reported RSV cases across the time series (online supplemental table S5). Subsequently, we restructured all four GAMs with ‘year’ input as a smoothing spline and formatted as a factorial, random effect to identify any deviant years in the overall climate or case time series (online supplemental table S5).

## Results

### Correlates of RSV infection by GAMs

**Figure 1:**
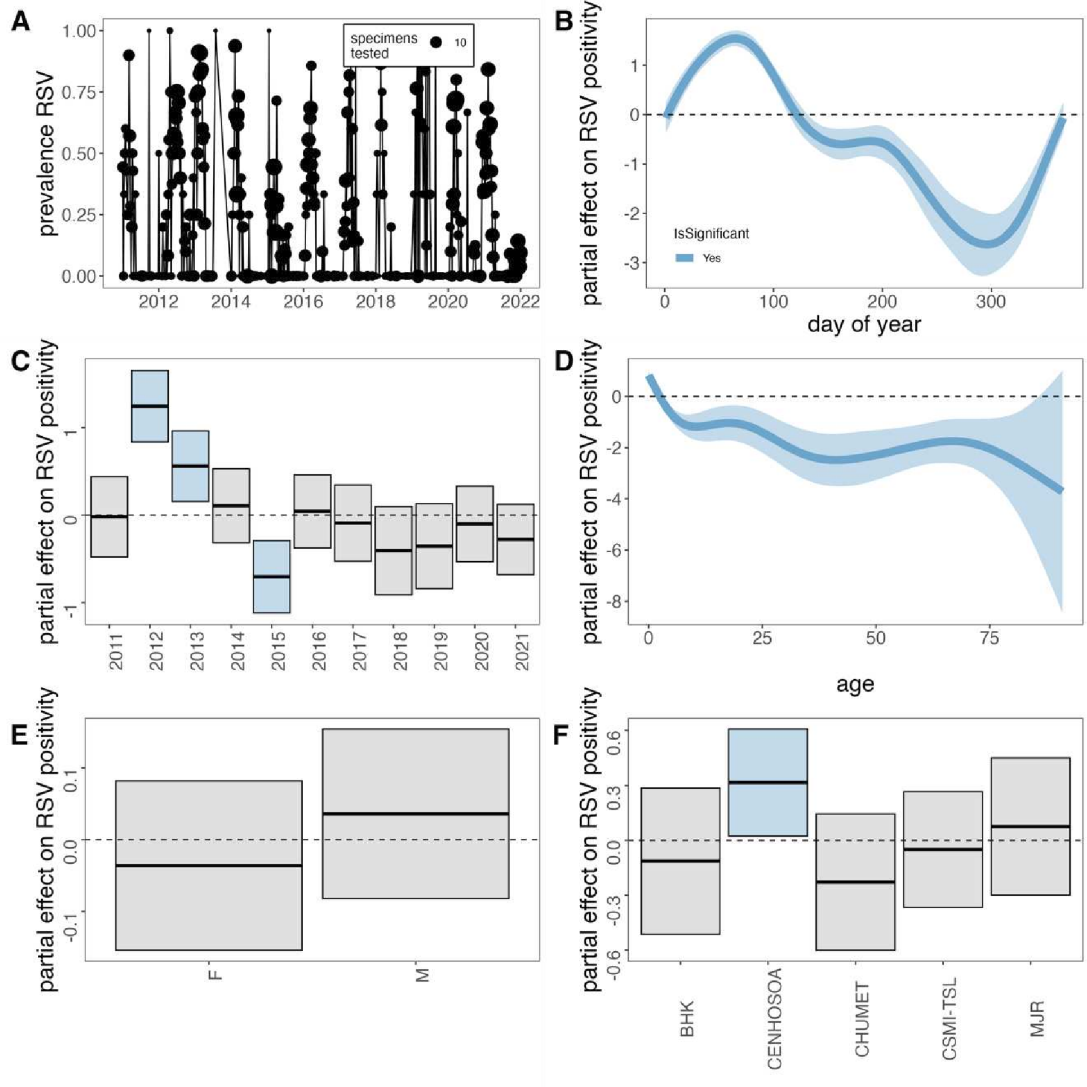
Generalised additive model (GAM) correlates of respiratory syncytial virus (RSV) positivity from influenza surveillance network testing in Antananarivo, Madagascar (2011–2021). (A) Time series of weekly RSV prevalence, where point size corresponds to the number of samples tested (total 3432) from January 2011 to December 2021. For GAM fitted to data with all available correlates (3242), partial effect sizes are shown for (B) day of year, (C) year, (D) age, (E) sex and (F) sampling locality on the probability of a sample testing positive for RSV infection. All predictors except sex contributed significantly to overall model performance. Shading corresponds to 95% CIs by SE. An effect is shaded in grey if the 95% CI crosses zero across the entire range of the predictor variable; in contrast, an effect is shaded in blue and considered ‘significant’ if the 95% CI does not cross zero. Metrics from GAM fits are reported in online supplemental table S1. BHK, Ostie Behoririka; CENHOSOA, Centre Hospitalier de Soavinandriana; CHUMET, Centre Hospitalier Universitaire Mère-Enfant Tsaralalàna; CSMI-TSL, Centre de Santé Maternelle et Infantile de Tsaralalana; MJR, Dispensaire Manjakaray.

From January 2011 to December 2021, a total of 3432 samples from reporting sites were screened for RSV. Of these, 989 (28.80%) tested positive for RSV infection (online supplemental figure S1). RSV prevalence varied considerably across the weekly time series, consistent with irregular reporting and seasonality (figure 1A).

We first investigated correlates of RSV positivity using GAMs fit to 3242 data points for which all possible metadata variables were reported. Our first GAM, in which we modelled year as a factorial random effect, performed slightly better than the second GAM modelling year as a numerical variable (online supplemental table S1); thus, we report the results of the best fit model only here. In this best fit GAM, we identified a significant effect of day of year, year, age and reporting site on RSV positivity; no significant association was identified for sex (figure 1; online supplemental table S1). In general, days 9–113 of each year (early January through late April) were significantly positively associated with RSV infection, consistent with records of the peak epidemic season for RSV in Madagascar (figure 1B). This relationship turned negative for the rest of the year, with a slight uptick at the end of December, as the onset of a new epidemic season approached. A few discrete years in the time series demonstrated significant deviations in reported RSV prevalence across the time series: years 2012 and 2013 were significantly positively associated with RSV infection, while year 2015 was negatively associated with RSV infection. Patients aged ≤2 years were additionally positively associated with RSV infection. Most other ages were negatively associated with RSV infection, though no significant directionality in association was identified in patients aged >85 years (figure 1D). The reporting hospital of CENHOSOA was also positively associated with RSV infection (figure 1F), likely a result of the overwhelming predominance of samples received from this locality. Day of year and annual patterns were largely recapitulated when modelling (in a third GAM) the entire dataset of 3432 samples without including age, sex and reporting hospital as predictors: in this model, days of year 7-111 were significantly positively associated with RSV infection, as were discrete years 2012, 2013 and 2019. As in the first GAM, year 2015 was also significantly negatively associated with RSV positivity (online supplemental table S1).

### Estimation of the age-structured FOI for RSV cases

**Figure 2:**
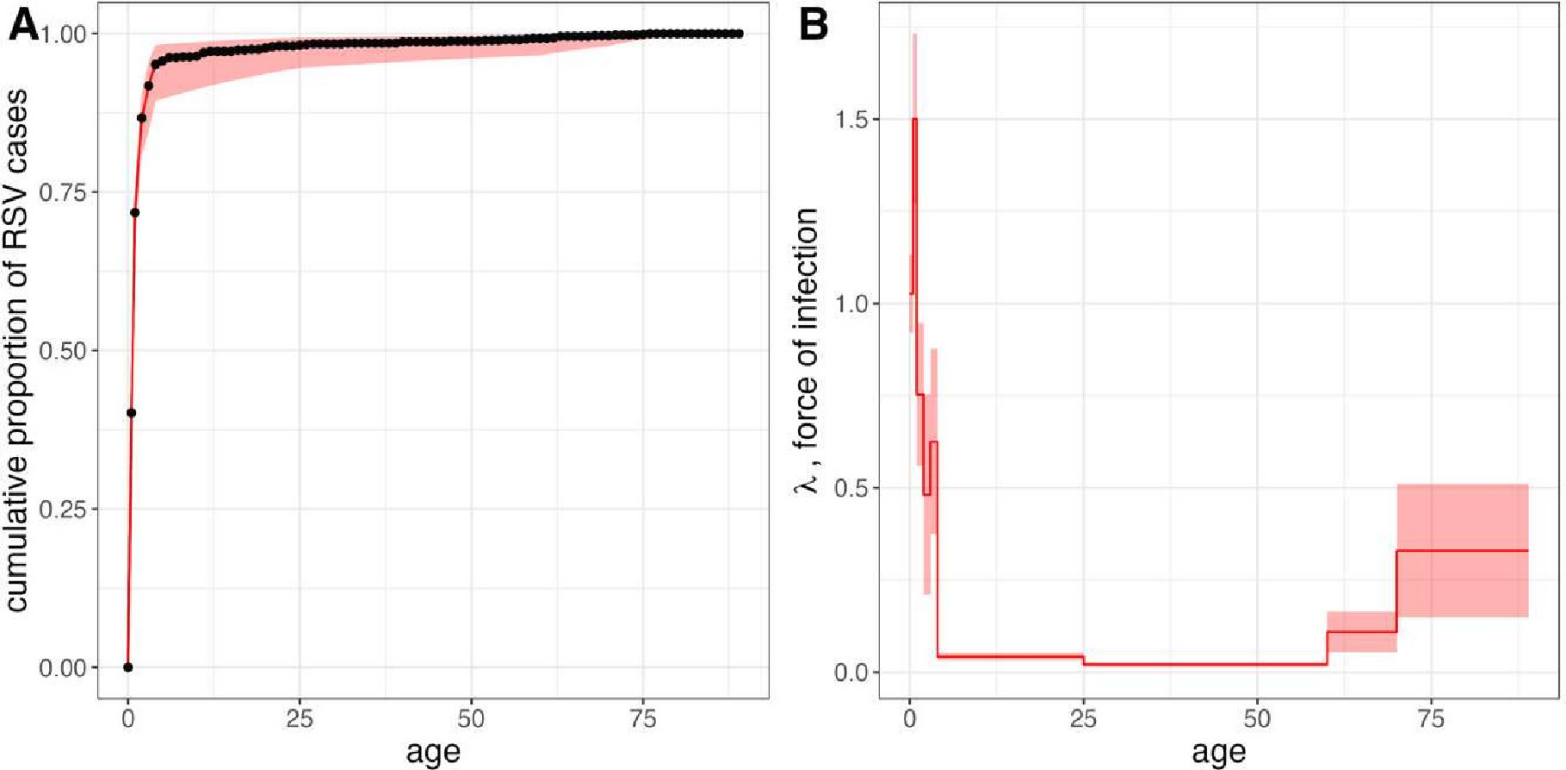
Catalytic model estimation of age-structured force of infection for respiratory syncytial virus (RSV) hospitalisations. (A) Cumulative proportion of hospitalised cases by age across 2011–2021 study period from all focal hospitals in Antananarivo (total=3259 data points). Black dots and connecting black line correspond to raw data binned across annual age brackets, with the first year of life separated into two age brackets (<0.5 and ≥0.5 years). Red line corresponds to model output from best fit force of infection (FOI) model, incorporating nine piecewise constant, age-specific hazards of infection across discrete age classes, as shown in (B). In both panels, red shading corresponds to 95% CI on FOI estimates computed from the Hessian matrix during model fitting; upper and lower CI bounds are used to project 95% CIs on modelled cumulative incidence in panel A. Comparisons of all models tested and exact FOI estimates from best fit model are reported in online supplemental table S2 .

Of the catalytic models tested, a model incorporating a piecewise, age-specific FOI across nine discrete age bins (with respective lower bounds of 0, 0.5, 1, 2, 3, 4, 25, 60 and 70 years) offered the best fit to the data (online supplemental table S2). Within this model framework, we estimated the highest FOIs in the two youngest cohorts (FOI=1.03 (0.92–1.13) for ages 0 to <0.5 years; FOI=1.50 (1.27–1.73) for ages 0.5 to <1 year) (figure 2). From this peak, FOI then decreased with increasing age across the first 4 years of life and sank to near-zero between ages 4 and 60 years (figure 2). FOI increased again in individuals aged 60 to <70 years (0.11 (0.05–0.16)) and individuals aged 70+ years (0.34 (0.15–0.51)). In general, we found improved model fit to the data when incorporating heightened specificity in λ(a) for CU5, as well as in individuals >60 years of age. Additional complexity in modelled age structure in older adolescents or young and middle-aged adults did little to improve model performance (online supplemental table S2). Consistent with the literature, these patterns suggest that the highest hazard of RSV infection is concentrated in the youngest and, to a lesser extent, oldest populations in Antananarivo.

### Seasonality of RSV transmission by TSIR modelling

**Figure 3:**
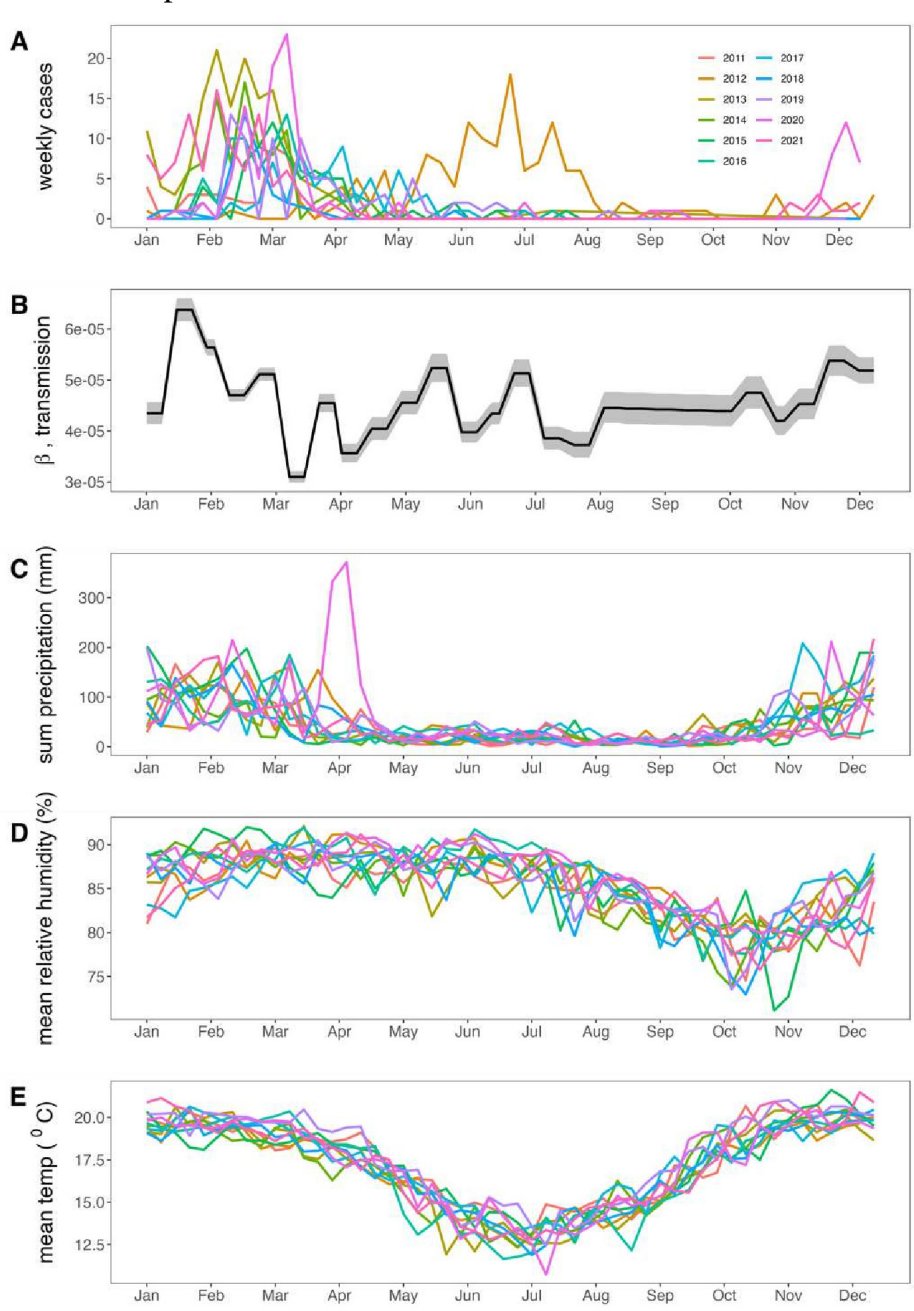
Seasonal trends in respiratory syncytial virus (RSV) infection and climate variables for Antananarivo, Madagascar (2011–2021). (A) Weekly total RSV cases across all reporting hospitals in our catchment area from January 2011 to December 2021, coloured by year of reporting as indicated in the legend. (B) Weekly RSV transmission rate, as estimated from time-series susceptible-infected-recovered (TSIR) model fitting across the year; shading corresponds to 95% CIs by SE from generalised linear model (GLM) fit. Raw weekly climate data for (C) total precipitation (mm), (D) mean relative humidity (%) and (E) mean temperature (°C) in Antananarivo across the time series, coloured by year of reporting as indicated in panel A.

We next binned reported RSV cases into weekly intervals across our time series for TSIR modelling. Consistent with results from the binomial GAMs, we observed from the raw data that weekly cases were highest in the first third of each year (January to April), excepting the year 2012, for which the epidemic peak was observed between May and August (figure 3A). Our efforts to reconstruct the population susceptible to RSV from publicly available birth rate data indicated significant under-reporting of RSV across the time series, with a mean estimated reporting rate of only 2.9% (online supplemental figure S2A–D). Nonetheless, the regression of cumulative reported cases against cumulative births was well supported (R^2^=0.98; online supplemental table S3) and produced a plausible estimate for the mean susceptible population of roughly 20% (online supplemental figure S2D). This quantity corresponds to an estimated R_0_=5 for RSV in our Antananarivo catchment area; coarse estimation of R_0_ using the inverse of the mean per capita birth rate (0.0327) and average age of infection (6.67) across the time series yields a comparable estimate of R_0_=4.58. Literature reported R_0_ values for RSV range from 1 to 9, with 3 as the most commonly cited statistic.^53^,^57^ Though our estimates are in the upper half of this range, R_0_=4.5–5 for RSV is not illogical given high birth rates and densely aggregated populations in Antananarivo.

Consistent with prior patterns observed in the GAMs (figure 1) and in the raw data (figure 3A), our fitted TSIR model estimated that RSV transmission peaked in January to February, slightly preceding cases, then declined throughout the rest of the year before climbing again in December (figure 3B; online supplemental figure S2E). As with susceptible reconstruction, our log-link linear regression to estimate transmission was well supported (McFadden pseudo-R^2^=0.64; online supplemental table S3) and successfully reconstructed the time series of cases following simulation (online supplemental figure S2F,G).

### Climate predictors of seasonal RSV transmission by GLMs

From the Antananarivo raw climate data, we observed that annual precipitation largely mirrored the seasonality of RSV, with most of the rainfall concentrated between January and the end of April and a slight increase in November and December leading up to the onset of each new year (figure 3C). Relative humidity also peaked in the early half of the year but remained high through June, with lows concentrated between September and November (figure 3D). Mean temperature followed the same broad pattern as the other climate variables but with a more gradual, sinusoidal annual cycle, with highs concentrated in the summer between November and March and lows between June and August (figure 3E).

**Figure 4:**
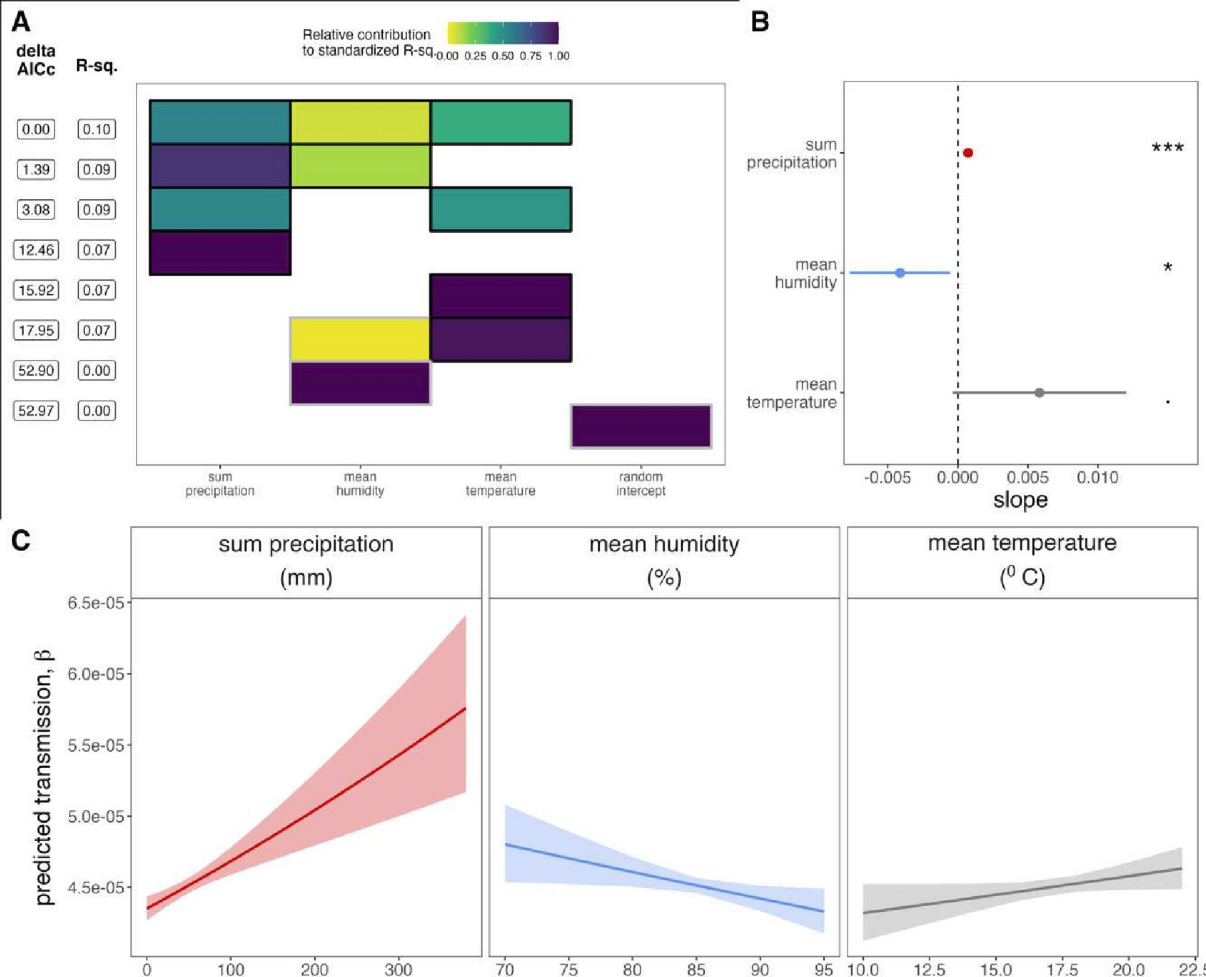
Climatic correlates of respiratory syncytial virus (RSV) transmission from generalised linear model (GLM) analysis. (A) Top eight GLMs using climate variables to predict RSV transmission from time-series susceptible-infected-recovered (TSIR), ranked by δAICc. Rows represent individual models and columns represent predictor variables. Cells are shaded according to the proportion of deviance explained by each predictor. Cells representing predictor variables with a p value significance level of <0.1 are outlined in black; all others are outlined in grey. (B) Effect size of significant climate correlates from best fit GLM (top row in panel A) on response variable of RSV transmission. 95% CIs by SE are shown as horizontal bars and corresponding p values are highlighted to the right for precipitation (***p<0.001), humidity (*p<0.05) and temperature (p<0.1). Statistical output is reported in online supplemental online supplemental table S4. (C) Predicted response of RSV transmission to a range of values for significant climate correlates from best fit GLM (top row in panel A); shading corresponds to 95% CIs by SE, computed for each effect size. δAICc, second-order Akaike information criterion.

Our top-performing GLM to evaluate the strength of association between climate and RSV transmission (as estimated from TSIR) included all three weekly Antananarivo climate variables (precipitation, humidity and temperature) as significant correlates of transmission, with precipitation contributing the most to the total deviance explained by the model (R^2^=0.10; figure 4A,B; online supplemental table S4). The second-best performing model included only precipitation and humidity as significant correlates of RSV transmission, again with a more pronounced effect for precipitation. In all cases, precipitation demonstrated a highly significant, positive correlation with the weekly RSV transmission rate (***p<0.001; figure 4B,C; online supplemental table S4). By contrast, relative humidity was significantly negatively associated with RSV transmission (*p<0.01; figure 4B,C; online supplemental table S4). Mean weekly temperature was positively correlated with transmission, but the association was considerably weaker than that observed for precipitation and only marginally significant (*p<0.1; figure 4B,C; online supplemental table S4).

### Interannual trends for climate variables and RSV cases by GAMs

GAMs fit to the 2011–2021 time series for all three Antananarivo climate variables demonstrated a significant, positive interannual trend in all cases, suggesting that precipitation, humidity and temperature have all increased across the past decade (precipitation: *p=0.05; humidity: *p=0.07; temperature: **p=0.004; online supplemental figure S3A–C; online supplemental table S5). Intra-annual trends captured in GAMs mimicked those previously described from observations of the raw data, with precipitation peaking at the beginning and end of each calendar year, relative humidity offset but still at its highest in the early half of the year, and temperature more evenly distributed with highs concentrated between November and March and lows from June to August (online supplemental figure S3A–C; figure 3C–E). In contrast to climate patterns, a Poisson GAM fit to the weekly time series of case counts demonstrated no significant interannual trend (p>0.1; online supplemental figure S3D; online supplemental table S5), after controlling for intra-annual cycling. Climate GAMs considering year as a factorial random effect indicated that year 2020 experienced significantly higher precipitation and humidity than average, while 2019 was found to be significantly warmer (online supplemental figure S3); by contrast, year 2011 was found to have lower-than-average humidity and years 2013 and 2016 to be significantly cooler than average (online supplemental figure S3). We identified no correspondence between those years exhibiting anomalous climate behaviour and those previously identified to have a higher-than-average RSV burden (2012 and 2013); RSV deviations already explored in figure 1 were largely confirmed by the Poisson GAM tested here (online supplemental figure S3).

## Discussion

In this study, we used a suite of modelling approaches to (1) characterise correlates for reported RSV infections, (2) quantify age structure in the force of RSV infection, (3) document seasonality in RSV transmission and (4) identify possible climatic drivers of this seasonality in the low-income setting of Antananarivo, Madagascar.

The results of our analysis of correlates of RSV transmission are consistent with those previously documented for this disease, both in Madagascar and elsewhere^7 9 58^: we find that reported hospitalisations for RSV are largely concentrated in very young patients (≤2 years of age) with a potential weaker association in elderly individuals (>60 years of age). Our quantification of the age-structured FOI for RSV indicates that the most intense transmission is focused on infants aged less than 1 year, with transmission intensity steadily decreasing to near-zero after 4 years of age, before rising again in elderly cohorts. Because we fit our FOI models to reported cases only, it is possible that less pathogenic RSV transmission may dominate among older children, resulting in asymptomatic infections, as has been previously suggested in the analysis of age-structured serological data from other regions.^59^ Our crude estimates for RSV R_0_, which we computed from the average age of infection and mean population-level birth rate, as well as from the mean susceptible population estimated from TSIR, additionally suggest that RSV transmission is elevated in Antananarivo, as compared with global averages.^53^,^57^ Again, our reliance on reported cases likely upwardly biases these calculations by over-representing more pathogenic strains or more vulnerable populations or both. Nonetheless, these estimates fall well within reported ranges for RSV R_0_,^53^,^57^ and a slightly elevated RSV transmission rate is not illogical, given Antananarivo’s young and densely aggregated population.

In addition to describing the magnitude and age structure of RSV transmission, our study also successfully quantifies intra-annual seasonality in RSV dynamics in Antananarivo. A seasonal concentration of RSV burden in the first half of the calendar year in Madagascar has been previously suggested based on more qualitative examination of the raw case data^8 9^; however, our study is the first to actually quantify RSV transmission, which slightly precedes cases. Our investigation of climatic correlates of the weekly transmission rate echoes recent work from the Northern Hemisphere which highlights a significant positive role for precipitation and negative role for humidity in driving RSV transmission dynamics.^17^ In addition, we identify a significant (though weaker) positive correlation between RSV transmission and temperature in Antananarivo. Because high temperatures and high precipitation are themselves correlated in tropical Madagascar’s rainy season, which spans October to March, it is possible that this muted contribution of temperature to RSV transmission dynamics may simply be an artefact of the precipitation driver, rather than a causative association of its own.

Our examination of interannual trends in the climate data suggests that precipitation, humidity and temperature have all increased across the past decade study period in Antananarivo, but that average RSV case load has remained constant. As high precipitation is associated with elevated RSV transmission rates, but high humidity is associated with lower RSV transmission rates in our dataset, it is possible that these climate drivers have had largely neutralising effects on the overall RSV burden throughout our time series. Nonetheless, our statistical models demonstrate a more pronounced effect of precipitation on the overall variation in observed RSV transmission, as has been reported in other tropical locations that demonstrate high average annual humidity with minimal variation across the year.^17^ Though there is no animal model available to empirically test the effects of climate on RSV transmission, experimental work in influenza has demonstrated that low humidity conditions favour transmission between guinea pigs, as a result of either increased survival of the virus and/or extended duration of virus circulation in aerosols under drier conditions.^60^ By contrast, though a population-level influence of precipitation has been observed on both influenza^60^,^62^ and RSV^18 63^ transmission, this effect has never been tested experimentally. Future studies empirically elucidating the mechanisms that underlie climate impacts on respiratory virus transmission would do much to validate our results. Future climate impacts on RSV transmission are likely to depend on the relative slope of each climate driver’s projected increase in a specific locality: over the past decade, precipitation has increased at a faster rate than humidity in tropical Antananarivo. If current trends hold, higher precipitation could drive future RSV transmission beyond the tempering effects of humidity.

Finally, our analysis highlights a few years in our decade-long time series that vary significantly from the overall trend, with higher-than-average or lower-than-average RSV-attributed infections and, in 1 year (2012), an irregular, off-season peak. Prior work in other systems has described off-season RSV epidemics attributed to irregular climate patterns,^64^ but we were unable to identify any association between deviant years in the RSV time series in Antananarivo and any observed climatic anomalies. Higher-than-average or lower-than average RSV burden in a given year could instead reflect the complex interplay between host immunity and circulating viral genotypes—both those of RSV itself, for which two major subtypes are known,^65^ and those of other respiratory infections known to induce some degree of anti-RSV cross-protective immunity.^66^ Prior sequencing efforts of Antananarivo RSV cases over the same time period indicate that year 2012 witnessed a transition in case load from the previously dominant RSV subtype B to RSV subtype A—genotype NA1, which was subsequently replaced by the introduction of the novel RSV subtype A—genotype ON1 in 2014.^62 67^ It is possible that the irregular seasonality of the RSV epidemic of 2012 reflects a lack of prior immunity in the Antananarivo CU5 population to RSV subtype B prior to turnover—though this hypothesis is impossible to test in the absence of additional viral genomic sequencing and paired subtype-specific serology. Off-season RSV transmission was well documented globally in 2020 and 2021 following relaxation of non-pharmaceutical interventions (NPIs) implemented to counter the initial spread of SARS-CoV-2.^68^ Intriguingly, our analysis did not recover any signature of aberrant RSV transmission for the year 2020 or 2021 in Antananarivo, suggesting that NPIs were largely unsuccessful at reducing respiratory virus transmission in this region during this time.^9^ Indeed, broad-scale serosurveys in 2020 and 2021 suggest that over 40% of the Antananarivo population was exposed to SARS-CoV-2 within the first 6 months of the pandemic,^69^ underscoring the relative ineffectiveness of NPIs on reducing respiratory disease burden.

Our study has several limitations. Our data were restricted to only a few reporting sites in Antananarivo, with most samples received from one sentinel site (CENHOSOA). This restricted geography somewhat impedes our ability to draw conclusions about broad trends in RSV dynamics across Madagascar. Additionally, as Madagascar is an isolated island in the Southwest Indian Ocean, understanding the drivers of RSV transmission in this locality may have limited potential for generalisability across the greater tropics. Influenza, for example, exhibits erratic seasonal dynamics in Madagascar that do not follow globally recognised patterns,^70^ limiting the extent to which transmission insights from Madagascar can be leveraged to guide policy interventions or surveillance elsewhere. Another limitation of our study is the necessarily arbitrary segregation of the population into discrete age classes for FOI estimation. While we attempted to consider all plausibly relevant delineations of age with respect to transmission, it is possible that we may have overlooked important dynamics hidden in untested hypotheses. Additionally, as mentioned, our dataset was limited to reported RSV cases only. More widespread prospective sampling of less virulent disease manifestations—either through molecular testing, or more feasibly, serology—would greatly enhance our inference into RSV transmission. Lastly, investigation of genome sequences would enable us to test hypotheses about the impact of virus genotype diversity on asynchronous and off-season dynamics.

In addition to challenges related to our own data and corresponding inference, we also faced challenges in the use of publicly available data for population sizes, birth rates and climate. In particular, we used government-reported values for the population served in the catchment area of each sentinel site in our analysis.^28 29^ Any uncertainty in these estimates could subsequently undermine downstream estimation of local birth rates and corresponding susceptible population reconstruction for input into TSIR. As a frequent limitation of TSIR approaches, we were additionally forced to extrapolate weekly birth rate data for our study population using very broad national-level, annual birth rate estimates from the World Bank. As seasonality in human birth rates has been shown to impact disease dynamics in other systems,^71^ lack of consideration of this effect in our analysis may somewhat undermine our inference into the transmission seasonality of RSV. More fine-scale birth data specifically tailored to our study region would do much to improve inference into infection dynamics. Finally, our climate records, which draw from Antananarivo at large, are (like our birth rate data) broad considering the highly localised (eg, within household) nature of the majority of RSV transmission.^72 73^ Despite these challenges, our modelling efforts recover plausible estimates for both the seasonality and R_0_ of reported cases of RSV in Antananarivo, suggesting that these uncertainties did not seriously impact our results.

All told, our study underscores the heavy morbidity and mortality burden that RSV presents to young children in Antananarivo and highlights an important role for climate in driving seasonal epidemics. Future changes in climatic parameters, particularly precipitation, are likely to impact RSV dynamics and may impact transmission, at least in the short term, in Madagascar. Introductions of new intervention strategies are greatly needed to mitigate RSV’s heavy mortality burden in low- and middle-income countries (LMICs)—especially considering possible intensification of burden in response to climate. RSV vaccines for older patients and pregnant mothers, as well as monoclonal antibody treatment for neonates, have recently become available in high-income countries.^74^ Despite projections of positive impact,^75^ these interventions have not yet reached the Global South, largely as a result of high cost barriers and lack of awareness of regional health authorities and communities regarding the burden of RSV. Shortages of human, financial and material resources, which jeopardise the provisioning of quality health services, are still a serious challenge in LMICs. As the majority of respiratory virus research is concentrated in the Global North, available interventions may additionally have limited efficacy in the Global South. In Madagascar, influenza vaccination is not included in the expanded programme on immunisation, and even if available, vaccine recommendations for both Northern and Southern Hemispheres are projected to be largely ineffective for Madagascar-specific influenza strains.^76^ Expansion of research into the dynamics and drivers of respiratory virus circulation in LMICs is thus greatly needed to inform the design of relevant therapeutics and guide the most effective plan for their introduction. Moreover, given that vaccines are not widely accessible and affordable for the whole population, efforts should be concentrated for vulnerable populations and resources allocated accordingly. Equitable global distribution of both infectious disease research and corresponding interventions need to be a major public health priority for the next decade.

## supplementary material

10.1136/bmjph-2024-001093 online supplemental file 1

## Data Availability

Data are available in a public, open access repository.

## References

[1] Andeweg SP, Schepp RM, van de Kassteele J (2021). Population-based serology reveals risk factors for RSV infection in children younger than 5 years. Sci Rep.

[2] OWi D (2019). Causes of death in children under five. WORLD.

[3] Shi T, McAllister DA, O’Brien KL (2017). Global, regional, and national disease burden estimates of acute lower respiratory infections due to respiratory syncytial virus in young children in 2015: a systematic review and modelling study. Lancet.

[4] Nair H, Nokes DJ, Gessner BD (2010). Global burden of acute lower respiratory infections due to respiratory syncytial virus in young children: a systematic review and meta-analysis. Lancet.

[5] Falsey AR, Hennessey PA, Formica MA (2005). Respiratory syncytial virus infection in elderly and high-risk adults. N Engl J Med.

[6] Haber N (2018). Respiratory syncytial virus infection in elderly adults. Med Mal Infect.

[7] Razanajatovo NH, Guillebaud J, Harimanana A (2010). Epidemiology of severe acute respiratory infections from hospital-based surveillance in Madagascar, November 2010 to July 2013. PLoS one.

[8] Rabarison JH, Tempia S, Harimanana A (2019). Burden and epidemiology of influenza- and respiratory syncytial virus-associated severe acute respiratory illness hospitalization in Madagascar, 2011-2016. Influenza Other Respir Viruses.

[9] Razanajatovo NH, Randriambolamanantsoa TH, Rabarison JH (2020). Epidemiological Patterns of Seasonal Respiratory Viruses during the COVID-19 Pandemic in Madagascar, March 2020–May 2022. Viruses.

[10] O’Brien KL, Baggett HC, Brooks WA (2019). Causes of severe pneumonia requiring hospital admission in children without HIV infection from Africa and Asia: the PERCH multi-country case-control study. The Lancet.

[11] Li Y, Wang X, Blau DM (2022). Global, regional, and national disease burden estimates of acute lower respiratory infections due to respiratory syncytial virus in children younger than 5 years in 2019: a systematic analysis. Lancet.

[12] Linssen RS, Bem RA, Kapitein B (2021). Burden of respiratory syncytial virus bronchiolitis on the Dutch pediatric intensive care units. Eur J Pediatr.

[13] Pinquier D, Crépey P, Tissières P (2023). Preventing Respiratory Syncytial Virus in Children in France: A Narrative Review of the Importance of a Reinforced Partnership Between Parents, Healthcare Professionals, and Public Health Authorities. Infect Dis Ther.

[14] Obolski U, Kassem E, Na’amnih W (2021). Unnecessary antibiotic treatment of children hospitalised with respiratory syncytial virus (RSV) bronchiolitis: risk factors and prescription patterns. J Glob Antimicrob Resist.

[15] Wambua J, Munywoki PK, Coletti P (2022). Drivers of respiratory syncytial virus seasonal epidemics in children under 5 years in Kilifi, coastal Kenya. PLoS One.

[16] Hogan AB, Glass K, Moore HC (2016). Exploring the dynamics of respiratory syncytial virus (RSV) transmission in children. Theor Popul Biol.

[17] Baker RE, Mahmud AS, Wagner CE (2019). Epidemic dynamics of respiratory syncytial virus in current and future climates. Nat Commun.

[18] Obando-Pacheco P, Justicia-Grande AJ, Rivero-Calle I (2018). Respiratory Syncytial Virus Seasonality: A Global Overview. J Infect Dis.

[19] Sapin G, Michault A, Simac C (1990). Seasonal trends of respiratory syncytial virus infections on Reunion Island gathering data among hospitalized children. Bull Soc Pathol Exot.

[20] Mathisen M, Strand TA, Sharma BN (2009). RNA viruses in community-acquired childhood pneumonia in semi-urban Nepal; a cross-sectional study. BMC Med.

[21] Matthew J, Pinto Pereira LM, Pappas TE (2009). Distribution and seasonality of rhinovirus and other respiratory viruses in a cross-section of asthmatic children in Trinidad, West Indies. Ital J Pediatr.

[22] Chew FT, Doraisingham S, Ling AE (1998). Seasonal trends of viral respiratory tract infections in the tropics. Epidemiol Infect.

[23] Heraud JM, Razanajatovo NH, Viboud C (2019). Global circulation of respiratory viruses: from local observations to global predictions. Lancet Glob Health.

[24] Rasolofonirina N (2003). Historique de la grippe à Madagascar. Arch Inst Pasteur Madag.

[25] Randrianasolo L, Raoelina Y, Ravololomanana L (2010). Surveillance sentinelle des fièvres à Madagascar. Rev Épidémiol Santé Publique.

[26] Rabarison JH, Rakotondramanga JM, Ratovoson R (2023). Excess mortality associated with the COVID-19 pandemic during the 2020 and 2021 waves in Antananarivo, Madagascar. BMJ Glob Health.

[27] Desa U (2018). The 2018 Revision of World Urbanization Prospects.

[28] INSTAT Madagascar (2021). Institut national de la statistique (instat) madagascar database rgph-3.

[29] Sentinel sites (2021). List of registered sentinel sites antananarivo, madagascar datasource.

[30] WHO (2013).

[31] WHO (2011).

[32] WHO (2002).

[33] Razanajatovo NH, Richard V, Hoffmann J (2008). Viral etiology of influenza-like illnesses in Antananarivo. PLoS one.

[34] NASA National aeronautics and space administration (nasa) database.

[35] Wood SN (2001). mgcv: GAMs and generalized ridge regression for R. R news.

[36] Muench H (1959). Catalytic Models in Epidemiology.

[37] Long GH, Sinha D, Read AF (2010). Identifying the age cohort responsible for transmission in a natural outbreak of Bordetella bronchiseptica. PLoS Pathog.

[38] Pomeroy LW, Bjørnstad ON, Kim H (2015). Serotype-Specific Transmission and Waning Immunity of Endemic Foot-and-Mouth Disease Virus in Cameroon. PLoS one.

[39] Heisey DM, Joly DO, Messier F (2006). The fitting of general force-of-infection models to wildlife disease prevalence data. Ecology.

[40] Grenfell BT, Anderson RM (1985). The estimation of age-related rates of infection from case notifications and serological data. J Hyg.

[41] Becker AD, Grenfell BT (2017). tsiR: An R package for time-series Susceptible-Infected-Recovered models of epidemics. PLoS One.

[42] Bjørnstad ON, Finkenstädt BF, Grenfell BT (2002). DYNAMICS OF MEASLES EPIDEMICS: ESTIMATING SCALING OF TRANSMISSION RATES USING A TIME SERIES SIR MODEL. Ecol Monogr.

[43] Grenfell BT, Bjørnstad ON, Finkenstädt BF (2002). DYNAMICS OF MEASLES EPIDEMICS: SCALING NOISE, DETERMINISM, AND PREDICTABILITY WITH THE TSIR MODEL. Ecol Monogr.

[44] Finkenstädt BF, Grenfell BT (2000). Time Series Modelling of Childhood Diseases: A Dynamical Systems Approach. J R Stat Soc Ser C Appl Stat.

[45] Baker RE, Mahmud AS, Metcalf CJE (2018). Dynamic response of airborne infections to climate change: predictions for varicella. Clim Change.

[46] Wesolowski A, Mensah K, Brook CE (2016). Introduction of rubella-containing-vaccine to Madagascar: implications for roll-out and local elimination. J R Soc Interface.

[47] Metcalf CJE, Bjørnstad ON, Ferrari MJ (2011). The epidemiology of rubella in Mexico: seasonality, stochasticity and regional variation. Epidemiol Infect.

[48] Bank TW (2023). The World Bank in Madagascar.

[49] Rm A (1991). Infectious diseases of humans. Aust J Public Health.

[50] Glass K, Xia Y, Grenfell BT (2003). Interpreting time-series analyses for continuous-time biological models—measles as a case study. J Theor Biol.

[51] Metcalf CJE, Bjørnstad ON, Grenfell BT (2009). Seasonality and comparative dynamics of six childhood infections in pre-vaccination Copenhagen. Proc R Soc B.

[52] Barton K, Barton MK (2015). Package ‘mumin.

[53] Reis J, Shaman J (2016). Retrospective Parameter Estimation and Forecast of Respiratory Syncytial Virus in the United States. PLoS Comput Biol.

[54] Reis J, Shaman J (2018). Simulation of four respiratory viruses and inference of epidemiological parameters. Infect Dis Model.

[55] Weber A, Weber M, Milligan P (2001). Modeling epidemics caused by respiratory syncytial virus (RSV). Math Biosci.

[56] White LJ, Mandl JN, Gomes MGM (2007). Understanding the transmission dynamics of respiratory syncytial virus using multiple time series and nested models. Math Biosci.

[57] Pitzer VE, Viboud C, Alonso WJ (2015). Environmental drivers of the spatiotemporal dynamics of respiratory syncytial virus in the United States. PLoS Pathog.

[58] Hall CB, Weinberg GA, Blumkin AK (2013). Respiratory syncytial virus-associated hospitalizations among children less than 24 months of age. Pediatrics.

[59] Nakajo K, Nishiura H (2023). Age-Dependent Risk of Respiratory Syncytial Virus Infection: A Systematic Review and Hazard Modeling From Serological Data. J Infect Dis.

[60] Shek L-C, Lee B-W (2003). Epidemiology and seasonality of respiratory tract virus infections in the tropics. Paediatr Respir Rev.

[61] Tamerius J, Nelson MI, Zhou SZ (2011). Global influenza seasonality: reconciling patterns across temperate and tropical regions. Environ Health Perspect.

[62] Tamerius JD, Shaman J, Alonso WJ (2013). Environmental predictors of seasonal influenza epidemics across temperate and tropical climates. PLoS Pathog.

[63] Omer SB, Sutanto A, Sarwo H (2008). Climatic, temporal, and geographic characteristics of respiratory syncytial virus disease in a tropical island population. Epidemiol Infect.

[64] De Silva LM, Hanlon MG (1986). Respiratory syncytial virus: a report of a 5-year study at a children’s hospital. J Med Virol.

[65] Peret TC, Hall CB, Schnabel KC (1998). Circulation patterns of genetically distinct group A and B strains of human respiratory syncytial virus in a community. J Gen Virol.

[66] Bhattacharyya S, Gesteland PH, Korgenski K (2015). Cross-immunity between strains explains the dynamical pattern of paramyxoviruses. Proc Natl Acad Sci USA.

[67] Eshaghi A, Duvvuri VR, Lai R (2012). Genetic variability of human respiratory syncytial virus A strains circulating in Ontario: a novel genotype with a 72 nucleotide G gene duplication. PLoS One.

[68] Eden J-S, Sikazwe C, Xie R (2022). Off-season RSV epidemics in Australia after easing of COVID-19 restrictions. Nat Commun.

[69] Razafimahatratra SL, Ndiaye MDB, Rasoloharimanana LT (2021). Seroprevalence of ancestral and Beta SARS-CoV-2 antibodies in Malagasy blood donors. Lancet Glob Health.

[70] Alonso WJ, Guillebaud J, Viboud C (2015). Influenza seasonality in Madagascar: the mysterious African free-runner. Influenza Other Respir Viruses.

[71] Martinez-Bakker M, Bakker KM, King AA (2014). Human birth seasonality: latitudinal gradient and interplay with childhood disease dynamics. Proc R Soc B.

[72] Hall CB, Geiman JM, Biggar R (1976). Respiratory syncytial virus infections within families. N Engl J Med.

[73] Munywoki PK, Koech DC, Agoti CN (2014). The source of respiratory syncytial virus infection in infants: a household cohort study in rural Kenya. J Infect Dis.

[74] Langedijk AC, Bont LJ (2023). Respiratory syncytial virus infection and novel interventions. Nat Rev Microbiol.

[75] Brand SP, Munywoki P, Walumbe D (2020). Reducing respiratory syncytial virus (RSV) hospitalization in a lower-income country by vaccinating mothers-to-be and their households. Elife.

[76] Guillebaud J, Héraud J-M, Razanajatovo NH (2017). Both hemispheric influenza vaccine recommendations would have missed near half of the circulating viruses in Madagascar. Influenza Other Respir Viruses.

